# The prognostic value of pain catastrophizing in health-related quality of life judgments after Total knee arthroplasty

**DOI:** 10.1186/s12955-018-0955-2

**Published:** 2018-06-18

**Authors:** Esther Yakobov, William Stanish, Michael Tanzer, Michael Dunbar, Glen Richardson, Michael J. L. Sullivan

**Affiliations:** 1 Department of Psychology, McGill University, 2001 McGill College, Room 1406, Montreal, Quebec H3A 1G1 Canada; 2 Department of Surgery, Dalhousie University, Halifax, Canada; 3 Department of Surgery, McGill University, Montreal, Canada

**Keywords:** Osteoarthritis, Pain catastrophizing, Health-related quality of life

## Abstract

**Background:**

Total knee arthroplasty (TKA) is a highly effective procedure that yields reductions in pain and disability associated with end stage osteoarthritis (OA) of the knee. Quality of life instruments are frequently used to gauge the outcomes of total knee arthroplasty (TKA). However, research suggests that post-TKA reductions in symptom severity may not be the sole predictors of quality of life post-TKA. The primary objective of the present study was to examine the prognostic value of catastrophic thinking in health-related quality of life (HRQoL) judgments in patients with severe OA after TKA.

**Methods:**

In this study we used a prospective cohort design to examine the value of pain catastrophizing in predicting HRQoL 1 year after TKA. Participants with advanced OA of the knee who were scheduled for TKA were recruited at one of three hospitals in Canada. The study sample consisted of 116 individuals (71 women, 45 men) who completed study questionnaires at their pre-surgical evaluation and 1 year after surgery. Hierarchical regression analysis was used to assess the unique contribution of pre-surgical pain catastrophizing to the prediction of post-surgical HRQoL judgments.

**Results:**

The results of the hierarchical regression equation revealed that the overall model was significant, F (9,106) = 8.3, *p* < 001, and accounted for 36.4% of the variance in the prediction of post-surgical physical component score of HRQoL. Pain catastrophizing was entered in the last step of the equation and contributed significant unique variance (β = −.35, *p* < .001) to the prediction of post-surgical physical component score of HRQoL above and beyond the variance accounted for by demographic variables, co-morbid health conditions, baseline HRQoL, and post-surgical reductions in pain, joint stiffness and physical disability.

**Conclusions:**

The current findings highlight the importance of pre-surgical catastrophic cognitions in influencing HRQoL judgments after TKA. The findings suggest that psychosocial interventions designed to reduce pain catastrophizing before TKA might contribute to better quality of life outcomes following surgery.

## Background

Total knee arthroplasty (TKA) has been shown to be effective for relieving pain, stiffness, mobility restrictions, and improving quality of life in patients with end-stage osteoarthritis (OA) of the knee [1–3]. Health-related quality of life (HRQoL) instruments are often used to gauge the success of TKA [4]. Research suggests that individuals’ perceptions of their quality of life might provide more meaningful information about their physical, social, and emotional well-being than medical status variables and other objective measures of symptomatology [5–9]. HRQoL instruments are particularly useful as markers of treatment effectiveness for conditions where complete eradication of symptoms is not considered to be a feasible treatment objective [4].

Review of the literature suggests that reduction in pain and improvement in function are strong predictors of patients’ reported improvement in HRQoL following TKA [10]. However, research also exists to indicate that improvement in condition specific symptoms such as knee pain and joint stiffness, does not always map directly onto patients’ ratings of improvement on indices of quality of life. For example, results of a large investigation indicated that while pain relief after TKA was reported by 98% of patients, only approximately 60% of patients reported significant improvement in HRQoL [11]. Moreover, research indicates that despite objective markers of surgical success, up to 30% of patients remain dissatisfied with improvement in quality of life outcomes after TKA [12, 13]. It has been proposed that in addition to disease-related variables, psychological variables might also play an important role in predicting HRQoL outcomes after TKA [11, 13]. To date systematic research examining psychological variables that can influence post-TKA HRQoL is lacking.

Catastrophic cognitions about pain have been shown to be associated with lower ratings of HRQoL in patients with various chronic pain conditions [14–17]. Pain catastrophizing has been broadly defined as a negative orientation towards actual or anticipated painful experiences [18]. Little research has been conducted to examine the association between pain catastrophizing and HRQoL in patients with severe OA of the knee. In one cross-sectional study, Somers and colleagues [19] reported a significant correlation between pain catastrophizing and a measure of HRQoL in patients with OA of the knee even after controlling for pain intensity. However, given the cross-sectional nature of the study, the direction of influence between pain catastrophizing and HRQoL remains uncertain. While it is possible that pain catastrophizing is contributing to negative HRQoL judgments, this relation may also simply reflect the psychological consequences of low HRQoL. In other words, low HRQoL may contribute to distress and exacerbate catastrophic cognitions. To date, the prospective relation between pre-surgical pain catastrophizing and post-surgical HRQoL judgments has yet to be systematically examined.

There are different pathways by which pain catastrophizing might impact on post-surgical HRQoL judgments. One possibility is that catastrophizing might compromise post-surgical recovery. Previous research has shown that high scores on pre-surgical catastrophizing are associated with persistent OA-specific pain severity and disability after TKA [20–22]. From this perspective, the negative impact of pain catastrophizing on post-surgical HRQoL would be entirely accounted for by problematic post-surgical outcomes. Another possibility is that pain catastrophizing might contribute to post-surgical HRQoL independent of its influence on surgical outcomes. The negative perceptual orientation of individuals high on pain catastrophizing might compromise coping strategies or lead them to focus excessively on their pain and avoid important life activities which, in turn, might have a negative impact on numerous domains of quality of life.

Demonstrating prospective relation between pain catastrophizing and post-surgical HRQoL judgments has important clinical implications. At present, efforts to maximize post-surgical HRQoL have primarily taken the form of improving medical or surgical management. Research showing independent contributions of psychological variables to post-surgical HRQoL would argue for the inclusion of psychological interventions to augment the impact of surgical procedures on post-surgical HRQoL. If HRQoL judgments are to be used as metrics to gauge the success and cost-effectiveness of surgical interventions for OA, there will be advantages to intervention approaches that target all possible variables that might influence post-surgical HRQoL.

Examining the contributions of pain catastrophizing to post-surgical HRQoL might also have important theoretical implications. Biopsychosocial models of surgical recovery are currently the dominant conceptual frameworks invoked to explain different recovery pathways. At present, however, these models have not been put forward with the degree of precision necessary to specify how different model components summate or interact to influence different recovery paths following surgery. Improving knowledge about the links between psychological and biological processes influencing post-surgical HRQoL might provide the conceptual foundation for the development of more effective interventions designed to maximize post-surgical HRQoL.

The purpose of the present study was to determine the value of pre-surgical pain catastrophizing in predicting HRQoL judgments after TKA. We tested the hypothesis that pre-surgical pain catastrophizing would be associated with lower post-surgical HRQoL independent of post-surgical reductions in pain, and physical disability and while controlling for demographic variables and co-morbid health conditions.

## Methods

### Participants

One hundred and sixteen individuals (71 women and 45 men) with severe OA of the knee who were scheduled for TKA at one of three hospitals in eastern Canada agreed to participate in this study. Participants ranged in age from 50 to 85 years. Fifty-two patients had TKA of the left knee, and 64 had TKA of the right knee. The majority of patients were married (85%) and had completed at least 12 years of education (90%).

### Measures

#### Pain catastrophizing

The Pain Catastrophizing Scale (PCS) consists of 13 items that assess thoughts and feelings related to the experience of pain [18]. Participants respond to items using a 5-point Likert-type scale with responses ranging from 0= “not at all” to 4= “all the time”. The scale has been shown to have high internal consistency (alpha =0.87), good test-retest reliability (*r* = 0.7–0.8), and to be associated with heightened experience of pain in patients with a wide range of health conditions including osteoarthritis [18, 20, 22–25]. The PCS yields subscale scores of rumination, helplessness, and magnification. In the present study, only the total score was used. In the present study, the alpha coefficient was 0.94.

#### Co-morbidities

Charlson Co-morbidity Index (CCI) [26] was used to assess the presence and severity of various co-morbid conditions that can affect TKA outcomes. The participants were asked to indicate the presence and severity of 13 different health conditions including hypertension, osteoarthritis of other joints, diabetes mellitus, and chronic obstructive pulmonary disease (COPD). The total score is calculated by summing the presence of different conditions indicated by the respondent [26].

#### Pain, disability, and stiffness

The Western Ontario and McMaster University Osteoarthritis Index (WOMAC) was used to evaluate disease specific outcomes; pain, joint stiffness and physical disability [27]. The WOMAC is a self-administered questionnaire that uses a 5-point Likert scale with responses ranging from 0 = “none” to 4 = “extreme”. Higher scores represent worse pain, stiffness, and disability. The WOMAC has been shown to be a valid and a reliable instrument for assessing disease specific outcomes in OA patients, and has been shown to be sensitive to changes in patients who underwent TKA [27–29].

#### Health related quality of life

Short-form health questionnaire (SF-12) was used to assess the physical and mental domains of quality of life. The SF-12 is a generic health status questionnaire that is derived from the SF-36, the original and longer version [30]. Scale scores range from 0 to 100, with lower scores indicating worse symptoms. It is a reliable and a valid instrument that is composed of two component scores; physical component score (SF-12 PCS) and mental component score (SF-12 MCS), and is used to assess the overall health-related-quality of life in individuals with pain related conditions including OA [31].

### Procedure

Patients in the current study were invited to participate at one of three hospitals where they were scheduled for TKA. They were informed that the research was concerned with the physical, psychological, and social determinants of recovery following surgery. To participate in the study, all patients provided written informed consent, and received $25 as compensation for completing the questionnaires. This study was approved by the Research Ethics Boards of the McGill University Health Centre, the Hôpital Maisonneuve-Rosemont, and the Capital Health Authority of Nova Scotia. Participants were asked to complete the questionnaires at the time of their pre-surgical evaluation (within 4 weeks of surgery) and at their one-year post-surgical follow-up. Surgeries were performed by 11 surgeons from three different hospitals. By clinical standards, all patients in the study were considered surgical successes.

### Data analysis

All measures (PCS, WOMAC, SF-12, Charlson Co-morbidity Index) showed a normal distribution with values of skewness below three and values of kurtosis below 10. Descriptive statistics were computed for sample characteristics on all pre- and post-surgical measures used in the study. To assess sex differences, independent sample t-tests were computed on all measures. Paired sample t-tests were computed to compare pre and post-surgical scores on the WOMAC and SF-12. Pearson correlations were used to examine the concurrent relations between pre-surgical measures of pain, disability, stiffness, mental and physical component scores of SF-12, and pain catastrophizing. A hierarchical regression was computed to assess the role of pre-surgical pain catastrophizing in the prediction of SF-12 PCS 1 year after surgery. Age, sex and BMI were entered in the first step. Co-morbid health conditions were entered in the second step. Baseline score of SF-12 PCS was entered in the third step. Change scores in disease specific variables (i.e., changes in raw scores from pre to post-WOMAC pain, disability, and stiffness) were entered in the fourth step. Pain catastrophizing was entered in the last step.

## Results

### Sample characteristics

Table 1 presents the means and standard deviations for all study variables. The mean age of the study sample was 67.1 years, with a range of 50 to 85 years. The mean BMI was 30.9, with a range of 20 to 45.2. The distribution of age, BMI, and pre surgical scores on measures of pain, disability, stiffness, SF-12 PCS, SF-12 MCS and pain catastrophizing were comparable to (within one standard deviation from the mean) those reported in previous studies with TKA patients [8, 24, 32–34]. Men and women did not differ significantly on any demographic or study variable except for post-operative SF-12 PCS where men reported higher scores (M = 45.6; SD =9.7) than women (M = 41.5; SD = 10.7) t (114) = − 2.1, *p* < .05. As presented in Table 1, pain catastrophizing was significantly correlated with pain severity, physical disability, SF-12 PCS and SF-12 MCS before surgery.

**Table 1:** Zero-order correlations among study variables before surgery

	M	SD	1	2	3	4	5	6	7	8	9	10
1. Sex												
2. Age	*67.1*	*8.2*	−.05									
3. BMI	*30.9*	*5.2*	−.02	.36*								
4. Comorbid	*2.9*	*1.4*	−.08	.27*	−.02							
5. PCS	*12.6*	*11*	−.10	−.10	.08	.19*						
6. PHQ-9	*6.7*	*7.0*	−.02	−.13	.01	.17	.51**					
7. Pain	*10.6*	*3.5*	−.07	−.28*	.21*	.13	.36**	.31*				
8. Function	*38.0*	*12.5*	.02	−.20*	.17	.20	.38**	.39**	.74**			
9. Stiffness	*4.7*	*1.5*	−.17	−.38**	.20*	.01	.30*	.31*	.68**	−.67**		
10. SF-12 PCS	*30.5*	*8.1*	.18	.01	−.12	−.24*	−.19*	−.25*	−.32**	−.42**	−.30*	
11. SF-12 MCS	*43.1*	*10.5*	.03	.06	−.06	−.27*	−.48**	−.57**	−.27*	−.27**	−.21*	.06

Note: *N* = 116. PCS = Pain Catastrophizing Scale; PHQ-9 = Patient Health Questionnaire; BMI = Pre-surgical Body Mass Index; Comorbid = comorbid health conditions; Pain = WOMAC Pain Score; Function = WOMAC Physical Function Score; Stiffness = WOMAC Stiffness Score; SF-12 PCS = SF-12 Physical component score of Quality of Life; SF-12 MCS = SF-12 Mental component score of Quality of Life

*p < .05, **p < .001

We also conducted zero order correlation analyses to examine the associations between age, BMI, and co-morbid health conditions with post-surgical component scores of SF-12. It was found that age, and BMI did not correlate with post-surgical component scores of SF-12. The number of co-morbid health conditions was associated with lower post-surgical SF-12 PCS (*r* = −.28, *p* < .05) and SF-12 MCS (*r* = −.20, p < .05).

### Changes between pre and post- surgical outcomes variables

Changes in pain, disability, stiffness, and HRQoL variables are presented in Table 2. As expected, there was a significant decrease in pain (− 7.2), *t* (115) = 18.9, *p* < .001, d = 1.8; stiffness (− 2.5) *t* (115) = 15.1, *p* < .001, d = 1.4; and disability (− 23.9), *t* (115) = 18.8, *p* < .001 d = 1.7 from the pre-surgical to post-surgical evaluation. Consistent with previous findings [33], significant post-surgical improvements were reported for physical (12.6) *t* (115) = − 13.3, *p* < .001, d = 1.2 but not mental component score of SF-12.

**Table 2:** Difference between pre- and post- surgical symptom severity and quality of life variables

Mean (SD)	Pre	Post	*P*	Cohen’s D
Pain	10.6 (3.5)	3.4 (3.4)	<.001	1.8
Disability	38.0(12.5)	14.1(11.4)	<.001	1.7
Stiffness	4.7 (1.5)	2.2 (1.5)	<.001	1.4
SF-12 PCS	30.5 (8.1)	43.1 (10.5)	<.001	1.2
SF-12 MCS	55.3 (10.4)	55.9 (7.9)	ns	.05

Note: *N* = 116. Pain = WOMAC Pain Score; Function = WOMAC Physical Function Score; Stiffness = WOMAC Stiffness Score; SF-12 PCS = SF-12 Physical component score of health-related quality of life; SF-12 MCS = SF-12 Mental component score of health-related quality of life

### Predicting HRQoL

Since the SF-12 MCS did not change as a result of surgery, the prospective relation between catastrophizing and HRQoL was tested only for the SF-12 PCS. The overall model was significant, *F* (9, 106) = 8.30, *p* < .001 and accounted for 41.3% of the variance (36.4% adjusted). As shown in Table 3, in the prediction of post-surgery SF-12 PCS, demographic variables entered in the first step failed to reach statistical significance. Co-morbid health conditions entered in the second step contributed 7% of variance in the prediction of post-surgery SF-12 PCS. Baseline SF-12 PCS entered in the third step contributed an additional 11.8% of variance to the regression equation. Changes in disease specific variables from pre- to post- surgery entered in the fourth step contributed 7.8% of the variance in the prediction of post-surgical SF-12 PCS. Pain catastrophizing entered in the last step accounted for additional 10.8% of the variance in the prediction of post-surgical SF-12 PCS. Examination of the standardized beta weights from the final regression equation indicated that only baseline SF-12 PCS (*β* = .37, *p* < .001) and pain catastrophizing (*β* = −.35, *p* < .001) contributed significant unique variance to the prediction of post-surgical SF-12 PCS.

**Table 3:** Regression analyses predicting post-surgical physical component of quality of life

	β	*p*	R^2^change
Dependent = Physical component score of health-related quality of life 1 year follow-up			
Step 1			
Age	.00	.98	
Sex	.07	.35	
BMI	−.05	.56	.04
Step 2			
Comorbid	−.13	.12	.07
Step 3			
SF-12 PCS T1	.37	.00	.12
Step 4			
Δ Function	.08	.48	
Δ Pain	.16	.17	
Δ Stiffness	.06	.54	.08
Step 5			
PCS	−.35	.00	.11

Note: *N* = 116. BMI = Body Mass Index; Comorbid = comorbid health conditions; Δ Pain = difference between pre and post-surgical WOMAC Pain Score; Δ Function = difference between pre and post-surgical WOMAC Physical Function Score; Δ Stiffness = difference between pre and post-surgical WOMAC Stiffness Score; SF-12 PCS T1 = SF-12 Physical component score of health-related quality of life at baseline; PCS = Pain Catastrophizing Scale

Standardized beta (β) is reported for the final step

## Discussion

The aim of the current study was to examine the prognostic value of pain catastrophizing in HRQoL judgments following TKA. Replicating the results of previous research with individuals with OA, pain severity and physical disability were associated with lower scores on a measure of HRQoL before surgery [35]. The results of the present study extend previous research by showing that high scores on pre-surgical pain catastrophizing predict lower scores on the physical component of HRQoL following TKA, independent of demographic variables, baseline SF-12 PCS, co-morbid health conditions, and reductions in pain severity, disability, and stiffness.

Gender, co-morbid health conditions, and obesity have been discussed as potential factors that can influence quality of life post-TKA [12]. In agreement with previous research, the results of the present study revealed that the number of co-morbid health conditions was significantly associated with lower post-surgical HRQoL [36]. Also consistent with previous research, the present study evidenced higher SF-12 PCS in men than in women after TKA [37]. Contrary to previous research findings, we did not find associations between BMI and post-TKA outcomes. This finding might be explained by a low variability in BMI in the present sample, with most scores (90.5%) falling in the overweight range. In agreement with previous research, the present study revealed that age was not associated with post-surgical HRQoL [38].

In recent years, there has been a growing interest in examining psychological contributions to health-related variables and recovery trajectories following TKA [39]. The finding that reduction in pain is not always directly proportional to patients’ reports of improved well-being has led researchers to search for other possible predictors of post-surgical HRQoL. Of interest in the present study was whether pain catastrophizing, measured before surgery, contributed to the prediction of post-surgical HRQoL. The pattern of findings suggests that pre-surgical pain catastrophizing is prospectively associated with lower post-surgical SF-12 PCS, above and beyond demographic variables, co-morbid health conditions and reductions in pain, and physical disability.

There could be several explanations for the present findings. Catastrophic cognitions about pain have been shown to interfere with fundamental neural processes related to pain perception through excessive attention to, anticipation of, and heighted emotional responses to pain [40, 41]. This hypervigilance to the threat value of pain might predispose high catastrophizers to avoidance of life activities perceived as potentially pain inducing [42]. Indeed, it has been shown that high catastrophizing in patients with OA undermined the willingness to engage in demanding physical activities, even though such activities are important for managing pain and disability [23]. Activity avoidance might also have a negative influence on HRQoL by depriving the individual from participating in important life activities, and from experiencing positive social interactions, potentially leading to physical deconditioning and social isolation. Additionally, pain catastrophizers are thought to possess l “pain schema” comprised of negative information about pain. Pain-related cognitive distortions might lead the individual to magnify the experience of pain, and adopt pessimistic beliefs about their ability to cope with pain [18]. It is also possible that a cognitive bias towards excessively negative information about pain may lead the individual to discount positive surgery outcomes (i.e., significant reduction in pain and disability) and report lower improvement in HRQoL despite significant reduction in symptom severity.

The results of hierarchical regression analysis revealed that demographic variables, pre-surgical HRQoL, co-morbid health conditions, reductions in symptom severity and physical disability, and pre-surgical pain catastrophizing accounted for approximately 36% of the variance in the prediction of post-surgical HRQoL, leaving 64% of the variance not accounted for by these variables. Other factors that have been associated with lower HRQoL include fatigue, mental health conditions, poor quality of sleep, lower socioeconomic status, and reduced social network [43–46]. It is possible that these variables might also contribute to lower HRQoL after TKA despite significant improvement in symptom severity, and independently from co-morbid health conditions and pain catastrophizing.

Osteoarthritis is the most common form of arthritis, and is the leading cause of disability in North America [47, 48]. With changing demographics, these figures are expected to increase substantially over the next decade. These figures are alarming, and signal that interventions designed to reduce the burden of OA are of increasing priority. At present, interventions designed to improve HRQoL in individuals with severe OA of the knee are primarily geared towards symptom reduction. Consistent with the results of previous investigations, the results of the present study suggest that a measure of pain catastrophizing should be considered as part of standard screening of individuals being considered for TKA. Targeting catastrophic thinking in patients at risk before surgery might be beneficial for improving post-surgical quality of life.

Psychosocial interventions combining cognitive-behavioural approaches to target catastrophic cognitions have been shown to be effective in promoting faster resumption of occupational activities and improved well-being in individuals with musculoskeletal injuries, low back pain, and fibromyalgia [49–52]. In vivo exposure techniques that enable patients to successfully experience and habituate to movements or activities that they have been avoiding have also been shown to decrease pain, and disability in patients with low back pain [53, 54]. It is possible that exposure techniques challenge and subsequently reduce catastrophic cognitions. Given a degree of success of these interventions in the context of persistent pain, adapting these interventions for individuals with OA holds promise.

The findings of the present study must be interpreted with caution. The study sample size was modest and replication with larger data sets is needed to bolster confidence in the validity and reliability of the present findings. The absence of process measures compromises the ability to make confident statements about the specific pathways by which pain catastrophizing influences HRQoL after TKA. Finally, although the discussion proceeded from the perspective that pre-surgical pain catastrophizing contributes to lower HRQoL after surgery, it is very likely that this association is bi-directional. In other words, while it is possible that pain catastrophizing contributed to lower HRQoL, it might also be possible that low HRQoL led to increased catastrophizing. For example, HRQoL might have been influenced by other factors not measured in the present study (i.e., co-morbid mental health conditions) that in turn might augment catastrophic cognitions. Despite these limitations, this was the first study to show that pre-surgical pain catastrophizing plays an important role in determining perceived HRQoL following TKA, above and beyond improvement in symptom severity, and independently from comorbid health conditions.

## Conclusion

The findings of the present study suggest that pain catastrophizing impacts on HRQoL despite significant reduction in pain, disability and stiffness after TKA, highlighting the importance for screening and targeting problematic cognitions before TKA. Future research is needed to determine whether screening and intervention for pain catastrophizing before surgery may improve HRQoL post-TKA.

## Abbreviations

BMI: Body mass index
CCI: Charlson Co-morbidity Index
HRQoL: Health-related quality of life
OA: Osteoarthritis
PCS: Pain Catastrophizing Scale
SF-12: Short-form health questionnaire
SF-12 MCS: Mental health component score of the short-form health questionnaire
SF-12 PCS: Physical health component score of the short-form health questionnaire
TKA: Total knee arthroplasty
WOMAC: Western Ontario and McMaster University Osteoarthritis Index
